# Electronic Data Capture Tools for Global Health Programs: Evolution of LINKS, an Android-, Web-Based System

**DOI:** 10.1371/journal.pntd.0002654

**Published:** 2014-04-10

**Authors:** Alex Pavluck, Brian Chu, Rebecca Mann Flueckiger, Eric Ottesen

**Affiliations:** Neglected Tropical Diseases Support Center (formerly Lymphatic Filariasis Support Center), The Task Force for Global Health, Atlanta, Georgia, United States of America; London School of Hygiene & Tropical Medicine, United Kingdom

## Introduction

The rapid expansion of mobile networks globally, coupled with the decreasing cost of mobile equipment [1], is allowing global health programs increasingly to utilize mobile- and cloud-based technology in their efforts to target important challenges to public health. Our initial electronic data collection system employed personal digital assistants (PDAs) [2], [3], but these proved to have significant cost and scalability limitations. The present report describes a second-generation, more efficient, cloud-based, smartphone-based system and the key elements that lead to its greater efficiency.

## The LINKS System

**Figure 1:**
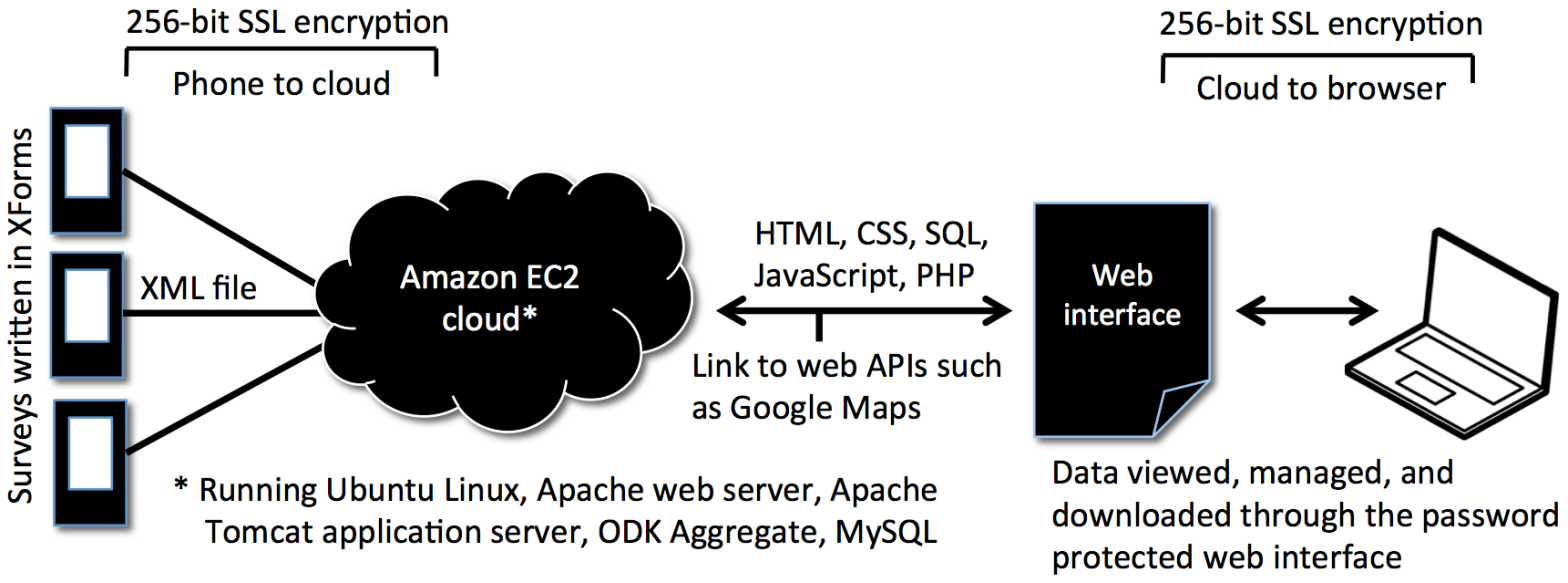
LINKS system technology overview. Data flow from the point of entry (cellular phones/tablets), to encrypted transmission to the cloud, to access and management of the data through a web interface.

While there are a number of tools available for data collection (EpiCollect, FormHub, EpiInfo, and others), these tools were not ideal for our purposes because of either license restrictions or other challenges. The starting point for the new mobile application, called the LINKS system (Figure 1), was the open source project Open Data Kit (ODK) [4], [5]. ODK allows the collection of a wide range of data using only the internal components of smartphone devices, such as the built-in GPS and the camera that can be used as a barcode scanner.

A server-based application (app) processes incoming data and writes those data to a database. A dynamic web interface was developed to present the collected data to the user in the form of tables, graphs, maps, and downloadable datasets. The system was deployed on Ubuntu Linux, running on Amazon.com's Elastic Cloud (AWS EC2, http://aws.amazon.com/ec2/) infrastructure. Geotrust secure certificates were installed to encrypt the data during transmission and between the user's browser and the server. Data are managed through a web interface or downloaded for offline use outside of the system.

The LINKS system was initially developed to address shortcomings of the earlier PDA-based data-capture systems and to support the interests of the Neglected Tropical Diseases (NTDs) community in employing an integrated approach to the NTDs using shared technical platforms. The LINKS system can

- support mobile technology running on a wide range of locally accessible hardware
- be used in both highly connected (internet) and connection-poor settings
- have a mechanism to deploy additional surveys to equipment already in the field
- be built entirely with industry-standard open source software to avoid costly licensing fees
- be cloud-based to allow for centralized management and increase scalability for large, highly dispersed projects

Since its launch in June of 2011, the LINKS system has been deployed to over 20 countries by multiple partner organizations (Table 1). Upon the completion of each project, both data collectors and project managers assessed its usability and the perceived benefits as well as the challenges to using the LINKS system (Table 2). This survey was administered to 30 individuals across academic and government organizations. Feedback was received from staff in Ethiopia, Tanzania, Kenya, Mozambique, Nigeria, Dominican Republic, and Indonesia. Additional feedback was received from implementing partners in the United States and United Kingdom.

**Table 1:** Implementation of LINKS system from June 2011–Aug 2013.

Organization	Country where deployed	Purpose of collection	Number of data collectors	Duration of project	Number of records collected
**The Task Force for Global Health (LFSC)**	Dominican Republic, Indonesia, Malaysia, Philippines, Sri Lanka, Tanzania, Togo, Tonga, Uganda, Vanuatu	Lymphatic filariasis operational research (OR)	50	1 year	>100,000
**Sight Savers International (DFID)**	Egypt, Ethiopia, Nigeria, Malawi, Yemen	Trachoma identification (Global Trachoma Mapping Project)	200	Ongoing (11 months)	>1,000,000
**Washington University (DOLF)**	Indonesia, Liberia, Republic of Congo	Lymphatic filariasis, Soil Transmitted Helminths (STH) OR	25	2 years	>76,000
**The Carter Center**	Nigeria	Trachoma, STH OR	40	Ongoing (4 months)	>200,000
**RTI International (USAID)**	Mozambique, Tanzania, Uganda	Lymphatic filariasis mapping, Trachoma mapping, OR	15	Ongoing (4 months)	>25,000
**Centers for Disease Control and Prevention (CDC)**	Haiti, Kenya, Mozambique, Nigeria, Tanzania	Lymphatic filariasis OR, Drug distribution coverage, Nutrition survey	30	various	>100,000
**University of Georgia (SCORE)**	Cote d'ivoire	Schistosomiasis OR	10	Ongoing (2 months)	>6,000
**Liverpool University**	Ethiopia	Lymphatic filariasis, Podoconiosis mapping	80	Ongoing (1 months)	>21,000
**IMA World Health**	Haiti	MDA Coverage and knowledge about treatments	20	3 months	>18,000

**Table 2:** Interview results.

Staff	Question	Cited feedback results
**Data collectors**	Do you find the LINKS system easier to use than the EDGE system?	+ Ease of entry
		+ New synchronization method (versus laptop connected synchronization)
		+ Local language surveys
	Do you find the LINKS system easier to use than standard paper forms?	− Lack of ability to modify form in the field
		+ Lack of data entry
		+ Automatic skip patterns
	Did you find the entry of data difficult using mobile devices?	− Gesture navigation as a challenge
		− Small keyboard
		− Challenge of scanning barcodes
**Program managers**	Do you find the online access to your data as a convent way to access, share, and manage data?	− Concerns over ownership
		− Concerns about needing internet access to access and manage data
	Do you find the real-time reporting useful to monitor the field work?	+ Capability of program monitoring while away from the project location.
	Would you want to use the LINKS system for other projects?	− Dissatisfaction in using shared system versus system running in country
		+ Speed at which data could be reviewed and used for implementation
	Did you find the deployment of LINKS difficult?	− Challenge of mobile data configuration
		+ Single device as much easier than EDGE multiple components

Cost savings were an immediately recognized benefit to deploying an app-based system that could run on any Android device (Table 3). The system was developed using open source applications and deployed on cost-effective cloud-based hosting. Acquisition costs of individual data collection devices were cut in half (and have continued to decrease), and shipping costs were also reduced by approximately half as there was not only less equipment (weight) being used but also no longer a need to ship equipment back to the central office for reprogramming before the next deployment. Training savings, too, were realized, because the system mirrors the existing paper forms and, compared to PDA systems, does not require extensive practice navigating multiple steps either to enter data or to send data to the server. Training costs are anticipated to be further reduced in the future as video-based training is introduced through websites such as YouTube (www.youtube.com).

**Table 3:** Cost comparison between previous PDA system and LINKS.

	Earlier, PDA-based system	Cost	LINKS System (smartphone/tablet)	Cost
**Data collection**	* HP iPAQ 211 PDA -	$200 USD	Android smartphone	$200 USD
	* Compact Flash GPS	$30 USD		
	* Secure Digital	$20 USD		
	* Laptop	$300 USD		
**Data transmission to central server**	Wired Internet connection following synchronization to laptop	$0	Cellular data service or Wi-Fi connect	$5–$10/month/phone ($0 if Wi-Fi used)
**Average equipment cost per project (assumes 5 data collectors)**		$4,000		$1,000
**Central server**	Microsoft Windows 2000 running Microsoft SQL Server 5.0	$10,000 USD software and hardware	Amazon Elastic Cloud Ubuntu running all open source software	$6,000 USD/year
**Typical number of days needed for training**	5		2	
**Replacement equipment**	Shipped from central office		Locally procured	
**Training time (per diem costs)**	5 days	∼$5,000 USD	2 days	∼$2,000 USD
**Redeployment**	Equipment needed to be shipped back to central office for reprogramming and software installation before shipping to next project site	∼$1,000 USD	Ship directly to next project site	∼$500 USD

Data quality is another important domain for evaluation. Routinely, the quality of the data has been assessed by reviewing the number of errors detected in the submitted data during the course of the project. The LINKS system automatically enforces point-of-source data checks (range checks, required variables, logical rules, etc.) to keep data errors to a minimum; however, detectable errors still exist. The largest LINKS project, the Global Trachoma Mapping Project (GTMP), is currently mapping the prevalence of trachoma in areas were trachoma infection is suspected. Over the next two years this project will evaluate over 4 million individuals in over 1,200 districts. Over the past 11 months, during which 1 million individuals were surveyed, this multinational project has seen a daily error rate of 0.14% (two errors/1,430 submissions). A future comparison of error rates between paper and other electronic data collection systems would be highly beneficial to validate the LINKS system further.

Finally, we evaluated the time between the end of data collection and implementation of results. A non-cloud-based system requires manual synchronization of data using local laptop computers, adding time and equipment. In contrast, a cloud-based system automatically synchronizes the data directly from the smartphones whenever connected to the internet, allowing data managers to identify and communicate issues with the field team during the collection of data. This allows data from all projects sites to flow from collection to implementation more rapidly. In the case of the GTMP, results take, on average, three days from the end of data collection to be included in public programmatic tools and for program implementation planning.

In addition to all that has been learned with this system over the past two years, it is important to note that mobile connectivity in remote areas (while sometimes still a challenge) is not an impediment to the implementation of these technologies.

## Challenges Remaining

Experiences with the LINKS system have also identified a number of persistent challenges to implementing a mobile-based system. Most notably, the essential network requirement of such a system can prove demanding in certain environments. While workarounds such as storing data locally until a connection is available are now feasible, many of these connectivity challenges will also likely solve themselves as the marketplace becomes increasingly dependent on widespread internet and cellular connectivity.

A further challenge with the cloud-based service model is the concern over data ownership, specifically, the acknowledgment that data is the sole property of the principal organization (e.g., national Ministries of Health) regardless of where the data are being stored. Similar efficiencies have been achieved from using a central cloud-based system in other technical services, such as e-mail and file storage. These services are accepted as the norm in personal use, and although it can be anticipated that national and global health data systems will move in this direction as well, this transition may be met with initial resistance.

The positive user feedback, combined with the cost-effective results from early deployment of the LINKS system, reinforces the drive to make continued development of electronic data collection systems and their rapid diffusion into daily use a priority for global health programs everywhere.
